# Plant Phenolics: Extraction, Analysis and Their Antioxidant and Anticancer Properties

**DOI:** 10.3390/molecules15107313

**Published:** 2010-10-21

**Authors:** Jin Dai, Russell J. Mumper

**Affiliations:** 1 Four Tigers LLC, 1501 Bull Lea Road, Suite 105, Lexington, Kentucky 40511 USA; Email: jdai2@uky.edu (J.D.); 2 Department of Pharmaceutical Sciences, College of Pharmacy, University of Kentucky, Lexington, Kentucky 40536, USA; 3 Division of Molecular Pharmaceutics, UNC Eshelman School of Pharmacy, University of North Carolina at Chapel Hill, Chapel Hill, North Carolina 27599, USA

**Keywords:** plant phenolics, extraction, analysis, antioxidant, anticancer

## Abstract

Phenolics are broadly distributed in the plant kingdom and are the most abundant secondary metabolites of plants. Plant polyphenols have drawn increasing attention due to their potent antioxidant properties and their marked effects in the prevention of various oxidative stress associated diseases such as cancer. In the last few years, the identification and development of phenolic compounds or extracts from different plants has become a major area of health- and medical-related research. This review provides an updated and comprehensive overview on phenolic extraction, purification, analysis and quantification as well as their antioxidant properties. Furthermore, the anticancer effects of phenolics *in-vitro* and *in*-*vivo* animal models are viewed, including recent human intervention studies. Finally, possible mechanisms of action involving antioxidant and pro-oxidant activity as well as interference with cellular functions are discussed.

## 1. An Introduction to Natural Phenolics

Phenolics are compounds possessing one or more aromatic rings with one or more hydroxyl groups. They are broadly distributed in the plant kingdom and are the most abundant secondary metabolites of plants, with more than 8,000 phenolic structures currently known, ranging from simple molecules such as phenolic acids to highly polymerized substances such as tannins. Plant phenolics are generally involved in defense against ultraviolet radiation or aggression by pathogens, parasites and predators, as well as contributing to plants’ colors. They are ubiquitous in all plant organs and are therefore an integral part of the human diet. Phenolics are widespread constituents of plant foods (fruits, vegetables, cereals, olive, legumes, chocolate, *etc.*) and beverages (tea, coffee, beer, wine, *etc.*), and partially responsible for the overall organoleptic properties of plant foods. For example, phenolics contribute to the bitterness and astringency of fruit and fruit juices, because of the interaction between phenolics, mainly procyanidin, and the glycoprotein in saliva. Anthocyanins, one of the six subgroups of a large group of plant polyphenol constituents known as flavonoids, are responsible for the orange, red, blue and purple colors of many fruits and vegetables such as apples, berries, beets and onions. It is known that phenolics are the most important compounds affecting flavor and color difference among white, pink and red wines; they react with oxygen and are critical to the preservation, maturation and aging of the wine.

**Figure 1:**
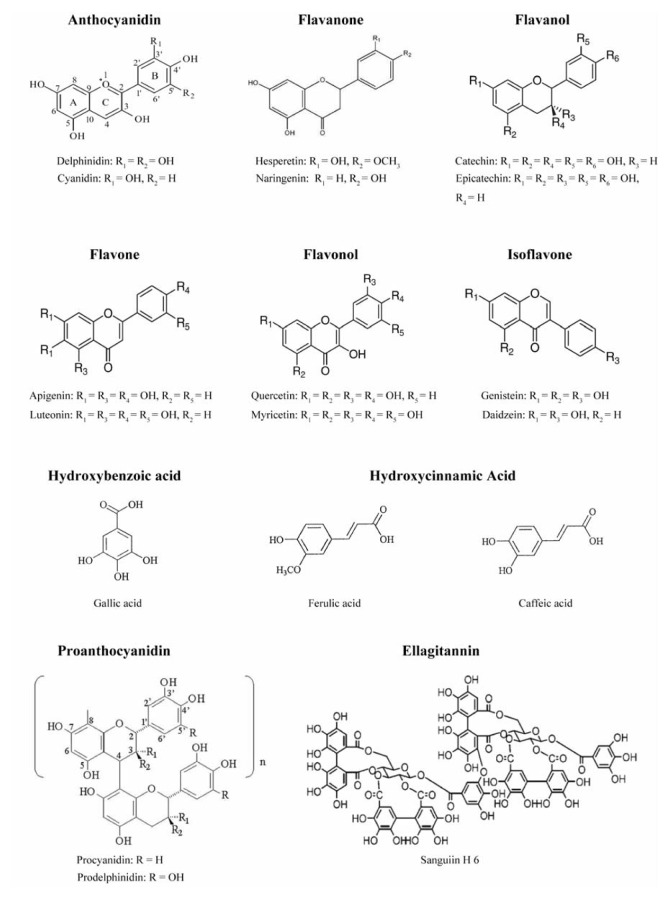
Structures of flavonoids, phenolic acids and tannins.

**Figure 2:**
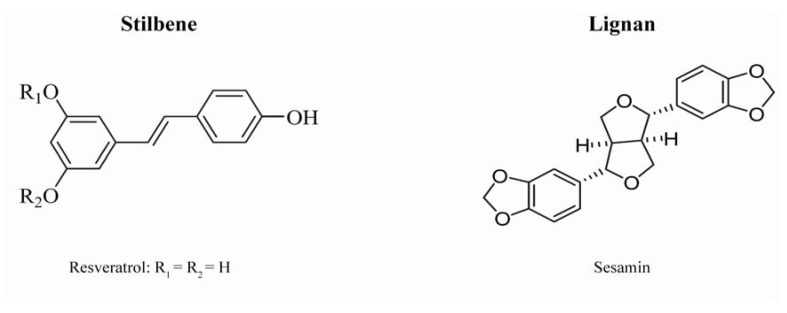
Structures of stilbenes and lignan.

Plant phenolics include phenolics acids, flavonoids, tannins (Figure 1) and the less common stilbenes and lignans (Figure 2). Flavonoids are the most abundant polyphenols in our diets. The basic flavonoid structure is the flavan nucleus, containing 15 carbon atoms arranged in three rings (C6-C3-C6), which are labeled as A, B and C. Flavonoid are themselves divided into six subgroups: flavones, flavonols, flavanols, flavanones, isoflavones, and anthocyanins, according to the oxidation state of the central C ring. Their structural variation in each subgroup is partly due to the degree and pattern of hydroxylation, methoxylation, prenylation, or glycosylation. Some of the most common flavonoids include quercetin, a flavonol abundant in onion, broccoli, and apple; catechin, a flavanol found in tea and several fruits; naringenin, the main flavanone in grapefruit; cyanidin-glycoside, an anthocyanin abundant in berry fruits (black currant, raspberry, blackberry, *etc.*); and daidzein, genistein and glycitein, the main isoflavones in soybean [1]. 

Phenolic acids can be divided into two classes: derivatives of benzoic acid such as gallic acid, and derivatives of cinnamic acid such as coumaric, caffeic and ferulic acid. Caffeic acid is the most abundant phenolic acid in many fruits and vegetables, most often esterified with quinic acid as in chlorogenic acid, which is the major phenolic compound in coffee. Another common phenolic acid is ferulic acid, which is present in cereals and is esterified to hemicelluloses in the cell wall [1]. 

Tannins are another major group of polyphenols in our diets and usually subdivided into two groups: (1) hydrolysable tannins and (2) condensed tannins. Hydrolysable tannins are compounds containing a central core of glucose or another polyol esterified with gallic acid, also called gallotannins, or with hexahydroxydiphenic acid, also called ellagitannins. The great variety in the structure of these compounds is due to the many possibilities in forming oxidative linkage. Intermolecular oxidation reactions give rise to many oligomeric compounds having a molecular weight between 2,000 and 5,000 Daltons [2]. Condensed tannins are oligomers or polymers of flavan-3-ol linked through an interflavan carbon bond. They are also referred to as proanthocyanidins because they are decomposed to anthocyanidins through acid-catalyzed oxidation reaction upon heating in acidic alcohol solutions. The structure diversity is a result of the variation in hydroxylation pattern, stereochemistry at the three chiral centers, and the location and type of interflavan linkage, as well as the degree and pattern of methoxylation, glycosylation and galloylation [3].

Despite their wide distribution, the health effects of dietary polyphenols have come to the attention of nutritionists only in recent years. Researchers and food manufacturers have become more interested in polyphenols due to their potent antioxidant properties, their abundance in the diet, and their credible effects in the prevention of various oxidative stress associated diseases [4]. The preventive effects of these second plant metabolites in terms of cardiovascular, neurodegenerative diseases and cancer are deduced from epidemiologic data as well as *in vitro* and *in vivo* [5,6,7,8] and result in respective nutritional recommendations. Furthermore, polyphenols were found to modulate the activity of a wide range of enzyme and cell receptors. In this way, in addition to having antioxidant properties, polyphenols have several other specific biological actions in preventing and or treating diseases.

## 2. Phenolic Sample Preparation and Characterization

### 2.1. Extraction

The extraction of bioactive compounds from plant materials is the first step in the utilization of phytochemicals in the preparation of dietary supplements or nutraceuticals, food ingredients, pharmaceutical, and cosmetic products. Phenolics can be extracted from fresh, frozen or dried plant samples. Usually before extraction plant samples are treated by milling, grinding and homogenization, which may be preceded by air-drying or freeze-drying. Generally, freeze-drying retains higher levels of phenolics content in plant samples than air-drying [9]. For example, Asami *et al*. showed that freeze-dried Marion berries, strawberries and corn consistently had a higher total phenolic content level compared with those air-dried [10]. However, drying processes, including freeze-drying, can cause undesirable effects on the constituent profiles of plant samples, therefore, caution should be taken when planning and analyzing research studies on the medicinal properties of plants [9].

Solvent extractions are the most commonly used procedures to prepare extracts from plant materials due to their ease of use, efficiency, and wide applicability. It is generally known that the yield of chemical extraction depends on the type of solvents with varying polarities, extraction time and temperature, sample-to-solvent ratio as well as on the chemical composition and physical characteristics of the samples. The solubility of phenolics is governed by the chemical nature of the plant sample, as well as the polarity of the solvents used. Plant materials may contain phenolics varying from simple (e.g., phenolic acids, anthocyanins) to highly polymerized substances (e.g., tannins) in different quantities. Moreover, phenolics may also be associated with other plant components such as carbohydrates and proteins. Therefore, there is no universal extraction procedure suitable for extraction of all plant phenolics. Depending on the solvent system used during exaction, a mixture of phenolics soluble in the solvent will be extracted from plant materials. It may also contain some non-phenolic substances such as sugar, organic acids and fats. As a result, additional steps may be required to remove those unwanted components. 

Solvents, such as methanol, ethanol, acetone, ethyl acetate, and their combinations have been used for the extraction of phenolics from plant materials, often with different proportions of water. Selecting the right solvent affects the amount and rate of polyphenols extracted [11]. In particular, methanol has been generally found to be more efficient in extraction of lower molecular weight polyphenols while the higher molecular weight flavanols are better extracted with aqueous acetone [12,13,14,15]. Ethanol is another good solvent for polyphenol extraction and is safe for human consumption [16]. In preparing anthocyanin-rich phenolic extracts from plant materials, an acidified organic solvent, most commonly methanol or ethanol, is used. This solvent system denatures the cell membranes, simultaneously dissolves the anthocyanins, and stabilizes them. However, care should be taken to avoid addition of excess acid which can hydrolyze labile, acyl, and sugar residues during concentration steps. To obtain the best yield of anthocyanin extraction, weak organic acids, such as formic acid, acetic acid, citric acid, tartaric acid and phosphoric acid, and low concentrations of strong acids, such as 0.5-3.0% of trifluoroacetic acid and < 1.0% of hydrochloric acid are recommended [17,18,19]. In addition, sulfured water has also been used as extraction solvent in seeking a reduction of the use of organic solvents as well as the cost of extraction [20].

The recovery of phenolic compounds from plant materials is also influenced by the extraction time and temperature, which reflects the conflicting actions of solubilization and analyte degradation by oxidation [21]. An increase in the extraction temperature can promote higher analyte solubility by increasing both solubility and mass transfer rate. In addition, the viscosity and the surface tension of the solvents are decreased at higher temperature, which helps the solvents to reach the sample matrices, improving the extraction rate. However, many phenolic compounds are easily hydrolyzed and oxidized. Long extraction times and high temperature increase the chance of oxidation of phenolics which decrease the yield of phenolics in the extracts. For example, conventional extraction and concentration of anthocyanins is typically conducted at temperatures ranging from 20 to 50°C [18], because temperatures > 70°C have been shown to cause rapid anthocyanin degradation [22]. Therefore, it is of critical importance to select efficient extraction procedure/method and maintain the stability of phenolic compounds. The conventional extraction methods such as maceration and soxhlet extraction have shown low efficiency and potential environmental pollution due to large volumes of organic solvent used and long extraction time required in those methods. A number of methods have been developed in recent years such as microwave, ultrasound-assisted extractions, and techniques based on use of compressed fluids as extracting agents, such as subcritical water extraction (SWE), supercritical fluid extraction (SFE), pressurized fluid extraction (PFE) or accelerated solvent extraction (ASE) were also applied in the extraction of phenolic compounds from plant materials.

Ultrasound-assisted extraction (UAE) is a potentially useful technology as it does not require complex instruments and is relatively low-cost. It can be used both on a small and large scale in the phytopharmaceutical extraction industry [23]. The mechanism for ultrasonic enhancement involves the shear force created by implosion of cavitation bubbles upon the propagation of the acoustic waves in the kHz range [24]. Collapse of bubbles can produce physical, chemical and mechanical effects [25], which resulted in the disruption of biological membranes to facilitate the release of extractable compounds and enhance penetration of solvent into cellular materials and improve mass transfer [23,26]. Recently, UAE has been widely used in the extraction of various phenolic compounds from different parts of plants such as leaves [27], stalks [28], fruits [29,30] and plant seeds [31]. A comparison study showed that UAE caused less degradation of phenolics and was a much faster extraction process in extraction of phenolic compounds from strawberries compared with other extraction methods including solid-liquid, subcritical water and microwave-assisted method [32].

Pressurized liquid extraction (PLE), also known under the trade name of accelerated solvent extraction (ASE), is a relative new technology for extraction of phytochemicals under high temperature and pressure. Benthin *et al*. [33] were among the first to conduct a comprehensive study on the feasibility of applying PLE to medicinal herbs after its emergence in the mid-1990s. In PLE, pressure is applied to allow the use as extraction solvents of liquids at temperatures greater than their normal boiling point. The combined use of high pressures (3.3-20.3 MPa) and temperatures (40-200°C) provides faster extraction processes that require small amounts of solvents (e.g., 20 min using 10–50 mL of solvent in PLE can be compared with a traditional extraction step in which 10–48 h and up to 200 mL are required) [34]. High temperature and pressure improves analyte solubility and the desorption kinetics from the matrices [35]. Therefore, extraction solvents including water which show low efficiency in extracting phytochemicals at low temperatures may be much more efficient at elevated PLE temperatures,. The use of water as an extraction solvent in PLE is the so-called subcritical water extraction (SWE). In SWE, water is heated up to 200°C and the change in the dielectric constant of the water with the temperature leads water to behave like an organic solvent. For example, the dielectric constant of water at 200°C is equal to 36 which is close to methanol [34]. Ju *et al*. showed that PLE (80–100°C) using acidified water was as effective as acidified 60% methanol in extracting anthocyanins from grape skins [36]. However, phenolic compounds are easily oxidized at high temperature so it is very important to prove that they will not degrade under the proposed PLE conditions [37]. In recent years, PLE has been successfully applied to the extraction of phenolic compounds from different plant materials such as grape seeds and skin [36,38,39], apples [40], spinach [41], eggplants [42] and barley flours [43]. Another technology using carbon dioxide as compressed fluid as extraction solvent is called supercritical and subcritical fluid extraction. Organic modifiers were added to increase the polarity of the fluid for extraction of phenolic compounds [44,45,46]. SFE is performed in the absence of both light and air; degradation and oxidation processes are significantly reduced in comparison with other extraction techniques. In general, all these compressed fluid-base extraction techniques are more environmental friendly procedures than other methods in reducing use of organic solvents (e.g., PLE), allowing extraction performed with nonpolluting, nontoxic solvents, such as water (e.g., SWE), supercritical CO_2_ fluid (e.g., SFE). However, due to the application of high pressure in these techniques, the requirements of instrumentation are high and the cost of these methods on the industrial scale is high which often outweigh the technical benefits.

Microwave-assisted extraction (MAE) is a process utilizing microwave energy to facilitate partition analytes from the sample matrix into the solvent. The main advantage of this technique is the reduced extraction time and solvent volume as compared to conventional extraction techniques [47]. It has been used for the extractions of some small-molecule phenolic compounds such as phenolic acids (e.g., gallic acid, ellagic acid) [48], quercetin [49], isoflavone [50] and trans-resveratrol [51] which were shown to be stable under microwave-assisted heating conditions at temperature up to 100°C for 20 min [52]. Phenolic compounds having a higher number of hydroxyl-type substituents (e.g., tannins) and those that are sensitive to elevated temperature (e.g., anthocyanins) may not suitable to be extracted by MAE due to degradation under MAE extraction conditions [52].

The extraction of phenolic compounds from plant materials may also be influenced by other factors such as solvent-to-solid ratio and the particle size of the sample. Increasing solvent-to-solid ratio was found to work positively for enhancing phenol yields [53,54]. However, an equilibrium between the use of high and low solvent-to-solid ratios, involving a balance between high costs and solvent wastes and avoidance of saturation effects, respectively, has to be found to obtain an optimized value [55]. Lowering particle size also enhances the yield of phenolic compounds [56,57]. To increase the release of bound phenolics, a number of enzymatic procedures involving the use of various mixed pectinolytic and cell wall polysaccharide degrading enzyme preparation in phenolic extraction have been described [58,59,60]. The particle size of the mashed samples was found to be a main factor to increase the enzyme action and extraction efficiency of phenolic compounds from samples in these enzyme-assisted extractions [61]. Besides enzymatic procedures, acid and alkaline treatments were found to be effective in releasing bound phenolics in phenolic extractions [62,63].

### 2.2. Purification and Fractionation

Plant crude extracts usually contain large amounts of carbohydrates and/or lipoidal material and the concentration of the phenolics in the crude extract may be low. To concentrate and obtain polyphenol-rich fractions before analysis, strategies including sequential extraction or liquid-liquid partitioning and/or solid phase extraction (SPE) based on polarity and acidity have been commonly used. In general, elimination of lipoidal material can be achieved by washing the crude extract with non-polar solvents such as hexane [64], dichloromethane [65], or chloroform [66]. To remove polar non-phenolic compounds such as sugars, organic acids, a SPE process is usually carried out. SPE is becoming popular since it is rapid, economical, and sensitive and because different cartridges and discs with a great variety of sorbents can be used. In addition, this technique can now be automated. C18 cartridges have been the most widely used in phenolic compound separation. After the aqueous sample is passed through preconditioned C18 cartridges, the cartridges are washed with acidified water to remove sugar, organic acids and other water-soluble constituents. The polyphenols are then eluted with absolute methanol [67] or aqueous acetone [64]. Further separation of phenolic compounds can be achieved by adjusting the pH of the sample as well as the pH and polarity of eluents. For example, Pinelo *et al*. adjusted the pH of dealcoholic wine sample to 7.0 and eluted phenolic acids with water in the first fraction [68]. Following this step, the C18 cartridge was acidified with 0.01 M HCl and nonpolymeric phenols such as catechins, anthocyanins, and flavonols were eluted with ethyl acetate. Finally, a mixture of water, acetone and methanol was used to elute the polymeric phenols. Other sorbents such as Amberlite XAD-2 [69], XAD-7 [70,71], XAD-16 [66], Oasis HLB [72,73] have also successfully been used to purify phenolic compounds in crude extracts or wine samples. A comparison of several SPE cartridge including Amberlite, silica-based C8, copolymer-based HLB, PH, ENV+ and MCX with silica-based C18 for the isolation of phenolic compounds in wine at low concentration showed that the proposed SPE method with HLB cartridge has a higher sensitivity, reproducibility and loading capacity than with C18 cartridge and HLB cartridge may be a good alternative for the C18 cartridge for the isolation of wine phenolic compounds [74]. 

Column chromatography has been also employed for fractionation of phenolic extracts. Although this method is often labor-intensive and solvent-consuming, it provides greater amounts of fractions for subsequent isolation and identification of pure substances. Typically-utilized column sorbents are RP-C18 [75], Toyopearl [76,77], LH-20 [76,77] and to a less extent polyamide resin [78]. Ethanol, methanol, acetone, and water and their combinations are commonly used as eluents. In particular, the isolation of proanthocyanidins (condensed tannins) is routinely carried out by employing Sephadex LH-20 column chromatography [79,80]. The crude extract was applied to the column which was washed with methanol or ethanol to elute the non-tannin substances followed by elution with acetone-water or alcohol-water to obtain proanthocyanidins. Using LH-20 column chromatography, methanol is more commonly used than ethanol to elute non-tannin compounds. Acetone-water is a much better solvent than ethanol-water to elute procyanidins from the column, especially polymeric procyanidins. In some cases, preparative-scale HPLC has also been used in polyphenol sample purification [81,82].

The classical liquid-liquid extraction procedure has been less commonly used because it is a tedious, highly time-consuming process with high solvent costs and low recoveries [83]. An example of sequential extraction was provided by extraction of phenolic compounds from tissues of cider apples [84]. The freeze-dried apple tissue powder was extracted sequentially with hexane (to remove lipids, carotenoids and chlorophyll), methanol (sugars, organic acids and phenolic compounds with low molecular weight) and aqueous acetone (polymerized polyphenols). As an alternative to liquid chromatography, Countercurrent Chromatography (CCC) has been developed as an effective technique for fractionation of various classes of phenolic compounds. CCC is a preparative all-liquid chromatographic technique based on partitioning of compounds between two immiscible liquid phases, a liquid stationary phase and a liquid mobile phase. Solutes are separated according to their partition coefficients between the two solvent phases based on their hydrophobicity. The big advantage of CCC is that it uses no solid matrix and the role of two liquid phases, namely, liquid stationary phase and mobile phase, can be switched during a run. Thus, there is no irreversible sample adsorption and the recovery is 100% [85]. Degenhardt *et al*. used high-speed countercurrent chromatography (HSCCC) for separation of anthocyanins in the pigment mixtures extracted from red cabbage, black currant, black chokeberry and roselle [86]. Anthocyanins were successfully fractionated based on their polarities into the biphasic mixture of *tert*-butyl methyl ether/*n*-butanol/acetonitrile/water (2:2:1:5, v/v/v/v) acidified with trifluoroacetic acid (TFA). Yanagida *et al.* demonstrated that HSCCC could be used for isolation of tea catechins and other food-related polyphenols such as procyanidins, phenolic acids and flavonol glycosides using *tert*-butyl methyl ether/acetonitrile/0.1% aqueous TFA (2:2:3, v/v/v) [87]. In addition, Krafczyk and Glomb employed Multilayer Countercurrent Chromatography (MLCCC) coupled with preparative High-Performance Liquid Chromatography (HPLC) to obtain pure flavonoids from Rooibos tea [88]. This method was able to isolate up to gram of material and to verify known polyphenol structures and discover previously unpublished ones [88]. 

### 2.3. Analysis and Quantification of Phenolics

Natural phenolics are of interest from many viewpoints (antioxidants, astringency, bitterness, browning reactions, color, *etc.*). Selection of the proper analytical strategy for studying phenolics in plant materials depends on the purpose of the study as well as the nature of the sample and the analyte [21]. The assays used for the analysis of phenolics are usually classified as either those measuring total phenolics content, or those quantifying a specific group or class of phenolic compounds. Quantification of phenolic compounds in plant extract is influenced by the chemical nature of the analyte, as well as assay method, selection of standards and presence of interfering substances [89]. 

Because of the heterogeneity of natural phenolics and the possible interference from other readily oxidized substances in the plant materials, it is not surprising that several methods have been used for determination of total phenolics and none are perfect [90]. Among such methods are the Folin-Denis method (FD), Folin-Ciocalteu method (F-C), permanganate titration, colorimetry with iron salts, and ultraviolet absorbance. In most cases, F-C has been found preferable as compared to the other methods [90]. The F-C assay relies on the transfer of electrons in alkaline medium from phenolic compounds to phosphomolybdic/phosphotungstic acid complexes to form blue complexes (possibly (PMoW_11_O_40_)^4−^) that are determined spectroscopically at approximately 760 nm [90,91]. Gallic acid is widely used as the comparison standard and values are usually compared as milligram of gallic acid equivalent per kilogram or liter of extract among samples. Owing to the general nature of the F-C chemistry, it is indeed a measure of total phenolics and other oxidation substrates. The other oxidation substrate present in a given extract sample can interfere the total phenolics measurement in an inhibitory, additive or enhancing manner [90,91]. The inhibitory effects could be due to the oxidants competing with F-C reagent and/or air oxidation after the sample is made alkaline. For this reason, the F-C reagent is added ahead of alkali [90]. Additive effects occur from unanticipated phenols, aromatic amines, high sugar levels or ascorbic acid in the samples. The additive effects can be measured before adding the alkali or by a more specific assay of a known interference and then subtracted from the F-C value [90]. Sulfites and sulfur dioxide which is a common additive for wine can cause enhancing effect [90]. Singleton *et al.* [90] discussed the effects of potential interference compounds and methods for correcting these factors. However, despite these disadvantages, the F-C assay is simple and reproducible and has been widely used for quantification of phenolic compounds in plant materials and extracts.

Anthocyanins are one of the six subgroups of the large and widespread group of plant phenolics known as flavonoids. While there are six common anthocyanidins, more than 540 anthocyanin pigments have been identified in nature [92]. The simplest assay for the quantification of anthocyanins as a group is based on the measurement of absorption at a wavelength between 490 nm and 550 nm, where all anthocyanins show a maximum. This band is far from the absorption bands of other phenolics, which have spectral maxima in the UV range [93]. However, by this method, anthocyanin polymerized degradation products produced by browning reactions are co-determined and lead to an overestimation of anthocyanin content. Therefore, an approach that differentiates anthocyanins from their degradation products is preferable. The pH differential method takes the advantage of the structural transformations of anthocyanin chromophore as a function of pH. By this method the absorption of the sample is measured at pH 1 (anthocyanins as colored oxonium salts) as well as at pH 4.5 (anthocyanins as colorless hemiketals). The anthocyanin degradation pigments do not exhibit reversible behavior with pH, and are thus excluded from the absorbance calculation [94]. In this method, calculation of monomeric anthocyanin concentration is usually based on the molecular weight (MW) and the molar extinction coefficient (ε) of either the main anthocyanin in the sample or cyanidin-3-glucoside, the most common anthocyanin in nature. For all quantification the MW and ε underlying the calculation should be given because the differences in the MW of the anthocyanins and the influence of the solvent on ε considerably distort the results [95]. For example, quantification as cyanidin-3-glucoside equivalents gave markedly lower results for berries containing mainly delphinidin and malvidin glycosides as compared with “real” values quantified based on corresponding standard compounds [71]. 

In a study of 20 food supplements containing extracts of blueberry, elderberry, cranberry and chokeberry, the total anthocyanin content (as determined as the cyanidin-3-glucoside equivalent) obtained with pH differential method were in good agreement with those obtained with an HPLC method [96]. In addition, a collaborative study where 11 collaborators representing academic, government and industrial laboratories analyzed seven fruit juice, beverage, natural colorants and wine samples demonstrated that total anthocyanin content can be measured with excellent agreement between laboratories using the pH differential method and the method has been approved as a First Action Official Method [97].

Anthocyanins are labile compounds and easily oxidized and condensed with other phenolics to form brown polymeric pigments. Somers and Evans developed a method based on the use of sodium sulfite, a bleaching reagent to determine the polymeric color and browning in wines [96]. Monomeric anthocyanins will combine with bisulfite to form a colorless sulfonic acid addition adduct while the polymeric anthocyanin degradation products are resistant to bleaching by bisulfite, as the 4-position is not available, being covalently linked to another phenolic compound. This method has been applied to a variety of anthocyanin-rich products and found to be extremely useful for monitoring the anthocyanin degradation and browning during processing and storage [95].

Different colorimetric methods are used to measure total proanthocyanidin (condensed tannin) content in plant samples. The proanthocyanidin assay is carried out in a butanol and concentrated hydrochloric acid (95:5, v/v) solution, where proanthocyanidins are autoxidized and cleaved to colored anthocyanidin monomer [98]. In the vanillin assay, condensation of resorcin- or phloroglucin- partial structure of flavonols with vanillin in acidic medium leads to the formation of colored carbonium ions [99]. Catechin, a monomeric flavanol, is often used as a standard. The same reaction mechanism as in the vanillin assay is used in the dimethylaminocinnamaldehyde (DMCA) assay, in which only the terminal units of the proanthocyanidins react with DMCA [100]. These methods of quantification are susceptible to the structure of the analytes as well as various external factors such as temperature, concomitant substances, solvent, presence of oxidants, *etc.* [101]. Thus, adaptation and validation of methods for different sample material are required. In addition, purification of proanthocyanidins before quantification has proven to be very supportive to minimize the interference and obtain reproducible results [101,102]. Over and above, these colorimetric methods for quantification of total proanthocyanidins are limited due to low yield because of the formation of side reaction products such as phlobatannins. Recently, a simple and robust method was developed and validated for the quantification of condensed tannins in grape extracts and red wine by precipitation with methyl cellulose, referred to as methyl cellulose precipitable tannin assay [103,104]. In this assay, condensed tannins are precipitated out in the sample by forming insoluble polymer-tannin complex with methyl cellulose and its concentration is determined by subtraction of phenolics contents in the sample monitored by measuring the absorbance at 280 nm before and after methyl cellulose treatment [103].

Hydrolysable tannins can be quantified by a number of approaches including the potassium iodate method, rhodanine method and sodium nitrite method. Of these, the potassium iodate method is most widely used. It is based on the reaction of methyl gallate, formed upon methanolysis of hydrolysable tannins in the presence of strong acids, with potassium iodate to produce a red chromophore with a maximum absorbance between 500 nm and 550 nm [105]. Similar as assays for proanthocyanidin quantification, the yield of this reaction also influenced by a number of factors such as the structure of the hydrolysable tannins, reaction time, temperature, other phenolics present in the sample, *etc.* The rhodanine method can be used for estimation of gallotannins and is based on determination of gallic acid in a sample subject to acid hydrolysis under conditions that must be anaerobic to avoid oxidation of the product [106]. On the other hand, the sodium nitrite assay is developed for quantification of ellagic acid in sample hydrolysate [107]. However, this assay requires large quantities of pyridine as a solvent which introduces a toxicity risk in the analysis procedure.

Since the characteristic reaction of tannins is their ability to precipitate protein, there are many methods developed to quantify tannins (both condensed and hydrolysable tannins) via protein binding. For example, tannins can be precipitated by a standard protein such as bovine serum albumin and the amount of tannin precipitated is assessed based on the formation of colored iron-phenolate complex in alkaline, detergent-containing solution. Detailed discussions on protein binding methods can be found in reviews by Hagerman and Butler [108,109].

In general, traditional spectrophotometric assays provide simple and fast screening methods to quantify classes of phenolic compounds in crude plant samples. However, due to the complexity of the plant phenolics and different reactivity of phenols toward assay reagents, a broad spectrum of methods is used for assay of the constituents, leading to differing and often non-comparable results. In addition to that, the methods are quite prone to interferences and consequently often result in over- or underestimation of the contents. Modern high-performance chromatographic techniques combined with instrumental analysis are the “state of art” for the profiling and quantification of phenolic compounds. Gas chromatographic (GC) techniques have been widely used especially for separation and quantification of phenolic acids and flavonoids. The major concern with this technique is the low volatility of phenolic compounds. Prior to chromatography, phenolics are usually transformed into more volatile derivatives by methylation, conversion into trimethylsilyl derivatives, *etc.* A detailed discussion on application of GC on analysis of phenolic acids and flavonoids was provided by Stalicas [110]. 

HPLC currently represents the most popular and reliable technique for analysis of phenolic compounds. Various supports and mobile phases are available for the analysis of phenolics including anthocyanins, proanthocyanidins, hydrolysable tannins, flavonols, flavan-3-ols, flavanones, flavones, and phenolic acids in different plant extract and food samples [13,111,112,113,114,115,116,117,118,119,120]. Moreover, HPLC techniques offer a unique chance to analyze simultaneously all components of interest together with their possible derivatives or degradation products [121,122]. The introduction of reversed-phase (RP) columns has considerably enhanced HPLC separation of different classes of phenolic compounds and RP C18 columns are almost exclusively employed. It was found that column temperature may affect the separation of phenolics such as individual anthocyanin [123] and constant column temperature is recommended for reproducibility [110]. Acetonitrile and methanol are the most commonly used organic modifiers. In many cases, the mobile phase was acidified with a modifier such as acetic, formic, and phosphoric acid to minimize peak tailing. Both isocratic and gradient elution are applied to separate phenolic compounds. The choice depends on the number and type of the analyte and the nature of the matrix. Several reviews have been published on application of HPLC methodologies for the analysis of phenolics [110,124,125,126].

**Figure 3:**
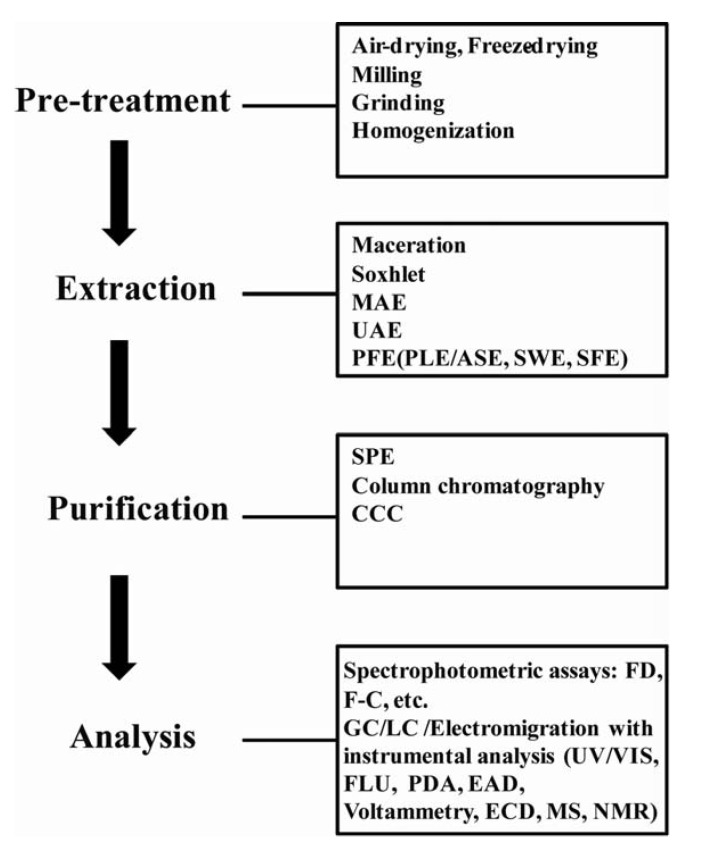
Strategies for preparation and characterization of phenolic samples from plant materials. Abbreviations: MAE, microwave-assisted extraction; UAE, ultrasound-assisted extraction; PFE, pressurized fluid extraction; PLE, pressurized liquid extraction; ASE, accelerated solvent extraction; SWE, subcritical water extraction; SFE, supercritical fluid extraction; SPE, solid phase extraction; CCC, countercurrent chromatography; FD, Folin-Denis method (FD), F-C, Folin-Ciocalteu method; GC, gas chromatography; LC, Liquid chromatography; FLU, fluorescence; PDA, photodiode array; EAD, electro-array detection; ECD, electrochemical detection; MS, mass spectrometric; NMR, nuclear magnetic resonance.

Given the intrinsic existence of conjugated double and aromatic bonds, every phenol exhibits a higher or lower absorption in ultraviolet (UV) or ultraviolet/visible (UV/VIS) region. Thus, the most common means of detection, coupled to LC, are UV/VIS, photodiode array (PDA), and UV-fluorescence detectors. PDA is the most prevalent method since it allows for scanning real time UV/VIS spectra of all solutes passing through the detector, giving more information of compounds in complex mixtures such as a plant crude extract. Other methods employed for detection of phenolic compounds include electrochemical detection (ECD) [127], voltammetry technique [128], on-line connected PDA and electro-array detection (EAD) [129], chemical reaction detection techniques [130], mass spectrometric (MS) [117,123,131] and nuclear magnetic resonance (NMR) detection [120,132]. MS and NMR detections are more of structure confirmation means than quantification methods.

Electromigration techniques including capillary electrophoresis (CE), capillary zone electrophoresis (CZE), and micellar electrokinetic chromatography coupled with UV, and to a less extent EC and MS detection are also employed for phenolics analysis [133].

## 3. Antioxidant Properties of Phenolic Compounds

Antioxidants are defined as compounds that can delay, inhibit, or prevent the oxidation of oxidizable materials by scavenging free radicals and diminishing oxidative stress. Oxidative stress is an imbalanced state where excessive quantities of reactive oxygen and/or nitrogen species (ROS/RNS, e.g., superoxide anion, hydrogen peroxide, hydroxyl radical, peroxynitrite) overcome endogenous anti-oxidant capacity, leading to oxidation of a varieties of biomacromolecules, such as enzymes, proteins, DNA and lipids. Oxidative stress is important in the development of chronic degenerative diseases including coronary heart disease, cancer and aging [134].

Recently, phenolics have been considered powerful antioxidants *in vitro* and proved to be more potent antioxidants than Vitamin C and E and carotenoids [135,136]. The inverse relationship between fruit and vegetable intake and the risk of oxidative stress associated diseases such as cardiovascular diseases, cancer or osteoporosis has been partially ascribed to phenolics [137,138]. It has been proposed that the antioxidant properties of phenolic compounds can be mediated by the following mechanisms: (1) scavenging radical species such as ROS/RNS; (2) suppressing ROS/RNS formation by inhibiting some enzymes or chelating trace metals involved in free radical production; (3) up-regulating or protecting antioxidant defense [139].

### 3.1. Phenolics as Free Radical Scavengers and Metal Chelators

Phenolic compounds (POH) act as free radical acceptors and chain breakers. They interfere with the oxidation of lipids and other molecules by rapid donation of a hydrogen atom to radicals (R):

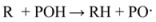
(1)

The phenoxy radical intermediates (PO·) are relatively stable due to resonance and therefore a new chain reaction is not easily initiated. Moreover, the phenoxy radical intermediates also act as terminators of propagation route by reacting with other free radicals:

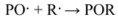
(2)

Phenolic compounds possess ideal structure chemistry for free radical scavenging activities because they have: (1) phenolic hydroxyl groups that are prone to donate a hydrogen atom or an electron to a free radical; (2) extended conjugated aromatic system to delocalize an unpaired electron. Several relationships between structure and reduction potential have been established as follows:

(1) For phenolic acids and their esters, the reduction activity depends on the number of free hydroxyl groups in the molecule, which would be strengthened by steric hindrance [140]. Hydroxycinnamic acids were found to be more effective than their hydroxybenzoic acid counterparts, possibly due to the aryloxy-radical stabilizing effect of the –CH=CH–COOH linked to the phenyl ring by resonance [136].

(2) For flavonoids, the major factors that determine the radical-scavenging capability [141,142] are:

(i) *the ortho-dihydroxy structure on the B ring*, which has the best electron-donating properties and confers higher stability to the radical form and participates in electron delocalization.
(ii) *the 2,3-double bond with a 4-oxo function in the C ring*, which is responsible for electron delocalization from the B ring.
(iii) *the 3- and 5-hydroxyl groups with the 4-oxo function in A and C rings*, which are essential for maximum radical scavenging potential.
(iv) *the 3-hydroxyl group is important for antioxidant activity.* The 3-glycosylation reduces their activity when compared with corresponding aglycones.

Quercetin is a flavonol that possess all of the factors described in (2). Anthocyanins are particularly reactive toward ROS/RNS because of their peculiar chemical structure of electron deficiency.

As an alternative antioxidant property, some phenolic compounds with dihydroxy groups can conjugate transition metals, preventing metal-induced free radical formation. The redox active metal ions such as Cu^+^ or Fe^2+^ interact with hydrogen peroxide (H_2_O_2_) through Fenton chemistry (as shown in reaction 3 below) to form hydroxyl radicals (·OH), which is the most reactive ROS known, being able to initiate free radical chain reactions by abstracting hydrogen from almost any molecule. Phenolic compounds with catecholate and gallate groups can inhibit metal-induced oxygen radical formation either by coordination with Fe^2+^ and enhancing autoxidation of Fe^2+^ (as shown in reaction 4 below), or the formation of inactive complex with Cu^2+^, Fe^2+^, or Cu^+ ^with relatively weaker interaction [143,144]. The attachment of metal ions to the flavonoid molecule can be 3’,4’-*o*-diphenolic groups in the B ring, 3,4 or 3,5-*o*-diphenolic groups, and the ketol structures 4-*keto*,3-hydroxy or 4-*keto*,5-hydroxy groups in the C ring [145,146]. It was also proposed that optimum metal-binding and antioxidant activity is associated with the structures which contain hydroxy-keto group (a 3-OH or 5-OH plus a 4-C = O), as well as a large number of catechol/gallol groups [145,147]. 


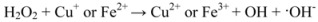
(3)


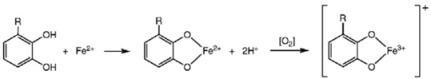
(4) [137]

Theoretically, these two antioxidant actions can cause a reduction of the steady state concentrations of free radicals and oxidant species. As a result, the subsequent oxidation of target molecules such as lipids, proteins and nucleic acids is diminished. Based on these potential capacities, extensive studies have demonstrated the antioxidant activities of natural phenolics, in general, in a myriad of biochemical and *ex vivo* systems [148], for example, in isolated low density lipoproteins (LDL), synthetic membrane, *ex vivo* human plasma, and cells in culture. In addition, mutual synergistic effects were also observed between different phenolic compounds or with other non-phenolic antioxidants [149] and it is generally accepted that a combination of phenolic or other antioxidants exert better antioxidant effect than pure individual compound.

### 3.2. Prooxidant Activity of Phenolic Compounds

It is worth noting that some phenolic antioxidants can initiate an autoxidation process and behave like prooxidants [141] under certain conditions. Instead of terminating a free radical chain reaction by reacting with a second radical, the phenoxy radical may also interact with oxygen and produce quinones (P = O) and superoxide anion (O_2_·^−^) as shown below [139]:
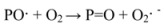
(5)

Nevertheless, transition metal ions could also induce prooxidant activity of phenolic antioxidants as demonstrated by the following reactions [150]:

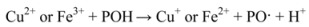
(6)

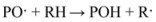
(7)

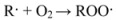
(8)

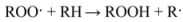
(9)

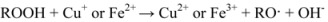
(10)


It was found that phenolic antioxidants behave like prooxidants under the conditions that favor their autoxidation, for example, at high pH with high concentrations of transition metal ions and oxygen molecule present. Small phenolics which are easily oxidized, such as quercetin, gallic acid, possess prooxidant activity; while high molecular weight phenolics, such as condensed and hydrolysable tannins, have little or no prooxidant activity [151]. It is necessary to consider the possible prooxidant effects of phenolics for *in vitro* antioxidant tests where great care should be taken in the design of experimental conditions. Moreover, because the biological conditions *in vivo* may differ dramatically from *in vitro* experiment, great caution must be taken when interpreting *in vitro* results and extrapolating to *in vivo* conditions.

### 3.3. Determination of Total Antioxidant Capacity (TAC) of Phenolic Extracts

Due to the chemical diversity of phenolic compounds and the complexity of composition in plant samples, it is costly and inefficient to separate each phenolic antioxidant and study it individually. Moreover, an integrated total antioxidant power of a complex sample is often more meaningful to evaluate the health benefits because of the cooperative action of antioxidants. Therefore, it is desirable to establish convenient screening methods for quick quantification of antioxidant effectiveness of phenolic extract samples. A variety of antioxidant assays such as Trolox equivalent antioxidant capacity (TEAC), oxygen radical absorbance capacity (ORAC), total radical-trapping antioxidant parameter (TRAP), ferric ion reducing antioxidant power (FRAP) and cupric ion reducing antioxidant capacity (CUPRAC) assays have been widely used for quantification of antioxidant capacity of phenolic samples from fruits and vegetables. The Folin-Ciocalteu antioxidant capacity assay (F-C assay, or total phenolics assay) is also considered as another antioxidant capacity assay because its basic mechanism is as oxidation/reduction reaction although it have been used as a measurement of total phenolics content for many years. On the basis of the chemical reaction involved, major antioxidant assays can be roughly classified as hydrogen atom transfer (HAT) and electron transfer (ET) reaction based assays although these two reaction mechanisms can be difficult to distinguish in some cases [150]. 

The HAT-based assays include ORAC and TRAP assays. These assays measure the capacity of an antioxidant to quench free radicals by hydrogen atom donation. The majority of HAT-based assays involve a competitive reaction scheme, in which antioxidant and substrate compete for thermally generated peroxyl radicals through the decomposition of azo compounds [150]. As an example of HAT-based assays, ORAC assay [152] employs a fluorescent probe (e.g., fluorescein) to compete with sample antioxidant for peroxyl radicals generated by decomposition of 2,2’-azobis (2-amidinopropane) dihydrochloride (AAPH). The fluorescence intensity is measured every minute at physiological conditions (pH 7.4, 37°C) to obtain a kinetic curve of fluorescence decay. The net area under the curve (AUC) calculated by subtracting the AUC of blank from that of the sample or standard (e.g., Trolox) and the TAC of sample is calculated as the Trolox equivalent based on a standard curve [150]. The ORAC method is considered to mimic antioxidant activity of phenols in biological systems better than other methods since it uses biologically relevant free radicals and integrates both time and degree of activity of antioxidants [153]. However, the method often requires the use of expensive equipment and it is usually a time-consuming process.

TEAC, F-C, FRAP and CUPRAC assay are ET-based assays. These assays measure the capacity of an antioxidant in reduction of an oxidant probe, which changes color when reduced [150]. The reaction is completed when the color change stops. The degree of color change is proportional to the concentration of antioxidant. The oxidant probes used are 2,2’-azinobis(3-ethylbenzothiazoline-6-sulfonic acid) radical cation (ABTS·^+^) in TEAC, Fe^3+^(2,4,6-tripyridyl-*s*-triazine)_2_Cl_3_ in FRAP and bis(neocuproine)Cu^2+^Cl_2_ in CUPRAC assays, respectively. The TEAC method is operationally simple, reproducible, and cost effective [154]. Most importantly, it can be applied in multiple media to determine both hydrophilic and hydrophobic antioxidant capacity of plant extracts since the reagent is soluble in both aqueous and organic solvent media [155]. As opposed to TEAC assay, FRAP assay measures ferric-to-ferrous reduction capacity of water-soluble antioxidants in acidic pH such as pH 3.6 [156].

It was proposed that procedures and applications for three assays, namely ORAC, F-C, and TEAC, be considered for standardization at the First International Congress on Antioxidant Methods held in Orlando, FL, in June 2004 [157]. It must be emphasized that these antioxidant assays measure the capacity of a sample only under defined conditions prescribed by the given method and strictly based on the chemical reaction *in vitro*, so the bioactivity of a sample cannot be reflected solely by these assays. In another words, the “total antioxidant capacity” of a particular sample cannot be truly measured by any of the assays because of the complexity of the chemistry of antioxidant compounds. For example, the total antioxidant capacity has to be able to reflect both lipophilic and hydrophilic capacity, and to reflect and distinguish hydrogen atom transfer, electron transfer, as well as transition metal chelation [157]. It is also very important to develop methods specific for each radical source for evaluating effectiveness of antioxidant compounds against various ROS/RNS such as O_2_·^−^, HO·, and ONOO^−^ to fully elucidate a full profile of antioxidant capacity [157].

## 4. Natural Phenolics and Cancer

Cancer is a multi-step disease incorporating environmental, chemical, physical, metabolic, and genetic factors which play a direct and/or indirect role in the induction and deterioration of cancers. Strong and consistent epidemiology evidence indicates a diet with high consumption of antioxidant-rich fruits and vegetables significantly reduces the risk of many cancers, suggesting that certain dietary antioxidants could be effective agents for the prevention of cancer incidence and mortality. These agents present in the diet are a very promising group of compounds because of their safety, low toxicity, and general acceptance [158]. Consequently, in the last few years, the identification and development of such agents has become a major area of experimental cancer research. Phenolic compounds constitute one of the most numerous and ubiquitous group of plant metabolites, and are an integral part of the human diet. It was found that in addition to their primary antioxidant activity, this group of compounds displays a wide variety of biological functions which are mainly related to modulation of carcinogenesis. Various *in vitro* and *in vivo* systems have been employed to determine the anticarcinogenic and anticancer potential of these natural phenolic compounds or extracts.

### 4.1. *In vitro* effects of phenolics

Phenolic extracts or isolated polyphenols from different plant food have been studied in a number of cancer cell lines representing different evolutionary stages of cancer. For example, berry extracts prepared from blackberry, raspberry, blueberry, cranberry, strawberry and the isolated polyphenols from strawberry including anthocyanins, kaempferol, quercetin, esters of coumaric acid and ellagic acid, were shown to inhibit the growth of human oral (KB, CAL-27), breast (MCF-7), colon (HT-29, HCT-116), and prostate (LNCaP, DU-145) tumor cell lines in a dose-dependent manner with different sensitivity between cell lines [66,159]. Katsube *et al*. compared the antiproliferative activity of the ethanol extracts of 10 edible berries on HL-60 human leukemia cells and HCT-116 cells and showed that bilberry extract was the most effective [160]. Ross *et al*. showed that the antiproliferative activity of raspberry extract in human cervical cancer (Hela) cells was predominantly associated with ellagitannins [161]. By comparing the phytochemical diversity of the berry extracts with their antiproliferative effectiveness, McDougall *et al*. suggested that the key component that related to the inhibition of cancer cell growth could be ellagitannins from the *Rubus* family (raspberry, arctic bramble, and cloudberry) and strawberry, whereas the antiproliferative activity of lingonberry was caused predominantly by procyanidins [162]. Similar results have also been reported in several cell system with wine extracts and isolated polyphenols (resveratrol, quercetin, catechin, and epicatechin) [163,164], tea extract and major green tea polyphenols (epicatechin, epigallocatechin, epicatechin-3-gallate, and epigallocatechin-gallate) [165,166,167], although the effective concentrations depend on the system and the tested substances. Other phenolic extracts or compounds intensely studies are from olives, legumes, citrus, apples, and also curcumin from spice turmeric. For example, soy isoflavone genistein can inhibit the growth of various cancer cell lines including leukemia, lymphoma, prostate, breast, lung and head and neck cancer cells [168]. Citrus flavonoids strongly inhibit the growth of HL-60 leukemia cells [169]. McCann *et al*. utilized established cell models of: genotoxicity (HT-29), invasion and metastatic potential (HT-115), and colonic barrier function (CaCo-2) to examine the effect of apple phenolic extract on key stages of colorectal carcinogenesis and found apple extract exert beneficial influence on all three carcinogenesis stages [170]. In addition, growth inhibitory effects of a number of polyphenols such as flavones (apigenin, baicalein, luteolin and rutin), flavanones (hesperidin and naringin) and sesame lignans (sesaminol, sesamin, and episesamin), which are not so extensively studied previously, have been examined in different cancer cell lines including colon [171], prostate [172,173], leukemia [174], liver [175], stomach, cervix, pancreas and breast [176]. 

### 4.2. *In vivo* Effects of Phenolics

In addition to *in vitro* studies on cancer cell lines, numerous *in vivo* experiments have also been performed to verify the antitumor efficacy of plant food-derived phenolic extracts or compounds with tumor incidence and multiplicity (e.g., number of tumors per animal) as endpoints [177,178,179,180]. The animal models commonly employed are either chemically, genetically, or ultraviolet light-induced tumor, as well as xenograft models, including colon, lung, breast, liver, prostate, stomach, esophagus, small intestine, pancreas mammary gland and skin tumors. As an example, Lala *et al*. investigated the chemoprotective activity of anthocyanin-rich extracts (AREs) from bilberry, chokeberry, and grape in Fischer 344 male rats treated with a colon carcinogen, azoxymethane (AOM) [181]. After 14 weeks, rats on ARE diets had significantly fewer colonic aberrant crypt foci (ACF) when compared with the control group. Moreover, rats fed bilberry ARE had 70% fewer large ACF compared with rats fed the control diet, indicating significant chemoprevention. Chokeberry-fed rats had a 59% reduction in large ACF, whereas the reduction was only 27% in rats fed grape ARE. The authors concluded that AREs from bilberry, chokeberry, and grape significantly inhibited ACF formation induced by AOM.

In another study by Ding *et al*. [182], cyanidin-3-glucoside (C3G), the major anthocyanin in blackberry, was investigated for the potential ability to inhibit 7,12-dimethylbenz[a]anthracene (DMBA)-12-*O*-tetradecanolyphorbol-13-acetate (TPA)-induced skin papillomas in animal skin model. Fourteen days following DMBA initiation, the dorsal skin of the mice was exposed to TPA in the presence or absence of C3G twice per week to cause promotion. The results showed that treatment of the animals with C3G (3.5 µM, topical application, twice/week) decreased the number of tumors per mouse at all exposure times. After 20 weeks of TPA promotion, a greater than 53% inhibition of papillomagenesis by C3G was observed. After 22 weeks, there were four tumors greater than 4–5 mm in diameter in the TPA-treated group, whereas no large tumors were found in the C3G plus TPA-treated group. In addition, they also tested the effects of C3G on human lung carcinoma (A549) xenograft growth and metastasis in athymic male nude mice. The results showed that C3G reduced the size of A549 tumor xenograft growth and significantly inhibited metastasis in nude mice. The authors concluded that C3G exhibits chemoprevention and chemotherapeutic activities by inhibiting tumor promoter-induced carcinogenesis and tumor metastasis *in vivo*.

The inhibition of tumorigenesis by tea preparations and its polyphenol constituents such as epigallocatechin-gallate (EGCG) and theaflavin have also been demonstrated in various animal models. However, caution must be taken when attributed the tumor inhibitory effect of tea to tea polyphenols in some animal models. For example, caffeine, a nonphenol constituent of tea, was found to contribute to the inhibitory effects of green and black tea on UVB-induced complete carcinogenesis [183], as well as the inhibition effects of black tea on lung tumorigenesis in F344 rats [184] to a significant extent. 

It is worth noting that the effectiveness of a phenolic extract in different organs is also dependent on the amount of its active constituents that can reach the target tissue. Therefore, the administration route and bioavailability factors of these extract constituents should be carefully considered when comparing their inhibition efficacy in different tumors. 

### 4.3. Human Intervention Studies Using Phenolics

Human intervention studies on potential health promoting or cancer preventive activity of polyphenol-rich food or food preparations have been conducted in healthy volunteers or individuals at high risk of developing cancer. Most studies have employed biomarkers reflecting antioxidant status or oxidative stress as endpoints, for example, plasma or serum antioxidant capacity, plasma malondialdehyde concentration, glutathione status, oxidative DNA damage in mononuclear blood cells (MNBCs), urinary 8-*epi*-prostaglandin F2α (8-Iso-PGF2) and 8-hydroxy-2’-deoxyguanosine (8-OHdG) concentration, *etc.*. Improvement of antioxidant status and/or protection against oxidative stress was observed in short term intervention studies (1 dose) with various polyphenol-rich food including fruit juices [185,186,187,188,189], red wines [190,191], chocolates [192,193,194] and fruits such as strawberries [190], as well as food preparations such as lyophilized blueberry powder [195], black currant anthocyanin concentrate [196], grape seed concentrate [197], dealcoholized [198] and lyophilized [190,199] red wines.

In a 6-month chemopreventive pilot study conducted by researchers from the Ohio State University, patients with Barrett’s esophagus (BE) were treated with 32 or 45 g (female and male, respectively) of freeze-dried black raspberries (FBRs) [200]. BE is a premalignant esophageal condition in which the normal stratified squamous epithelium changes to a metaplastic columnar-lined epithelium and is underscored by the fact that it increases the risk for the development of esophageal adenocarcinoma, a rapidly increasing and extremely deadly malignancy by 30- to 40-fold [201]. Their results suggested that daily consumption of FBRs reduced the urinary excretion of 8-Iso-PGF2 and 8-OHdG, among patients with BE indicating reduced oxidative stress [200]. The same group of researchers also investigated a novel mucoadhesive gel formulation for local delivery of FBRs to human oral mucosal tissues [202]. The results indicated that a gel formulation was well-suited for absorption and penetration of anthocyanins into the target oral mucosal tissue site as evidenced by detectable blood levels within 5 min after gel application and the greater penetration of anthocyanins into tissue explants was observed in berry gels with a final pH of 6.5 *versus* pH 3.5 [202]. Furthermore, the effects of the 10% (w/w) FBR gel formulation was examined clinically on oral intraepithelial neoplasia (IEN), a recognized precursor to oral squamous cell carcinoma [203,204]. It was found that topical FBR gel application (0.5 g applied four times daily for six weeks) was well tolerated in all the 27 trial participants [203]. Results from this clinical trial showed that FBR gel topical application significantly reduced loss of heterozygosity (LOH) indices at chromosomal loci associated with tumor suppressor genes [203], uniformly suppressed gene associated with RNA processing, growth factor recycling and inhibition of apoptosis and significantly reduced epithelial COX-2 levels in human oral IEN lesions [204]. In addition, it was found gel application also reduced microvascular density in the superficial connective tissues and induced genes associated with keratinocyte terminal differentiation in a subset of patients [204].

A recent study evaluated the effects of anthocyanin/polyphenolic-rich fruit juice consumption on antioxidant status in hemodialysis patients that are facing an elevated risk of cancer, arteriosclerosis, and other diseases, ascribed in part to increased oxidative stress [205]. In this pilot intervention study, 21 hemodialysis patients consumed 200 mL/day of red fruit juice (3-week run-in; 4-week juice uptake; 3-week wash-out). Weekly blood sampling was done to monitor DNA damage (comet assay +/− formamidopyrimidine-DNA glycosylase enzyme), glutathione, malondialdehyde, protein carbonyls, Trolox equivalent antioxidant capacity, triglycerides, and DNA binding capacity of the transcription factor nuclear factor-kappa B (NF-κB). Results show a significant decrease of DNA oxidation damage (P < 0.0001), protein and lipid peroxidation (P < 0.0001 and P < 0.001, respectively), and NF-κB binding activity (P < 0.01), and an increase of glutathione level and status (both P < 0.0001) during juice uptake. The authors attributed this reduction in oxidative (cell) damage in hemodialysis patients to the especially high anthocyanin/polyphenol content of the juice. The authors concluded that consumption of antioxidant berry juices appears to be a promising preventive measure to reduce chronic diseases such as cancer and cardiovascular disease in population subgroups exposed to enhanced oxidative stress like hemodialysis patients [205].

### 4.4. Mechanism of Action of Phenolics

Cancer development is a multistage process that involves a series of individual steps including initiation, promotion, progression, invasion and metastasis. Tumor initiation begins when DNA, in a cell or population of cells, is damaged by exposure to carcinogens, which are derived from three major sources: cigarette smoking, infection/inflammation, and nutrition/diet [206].

**Figure 4:**
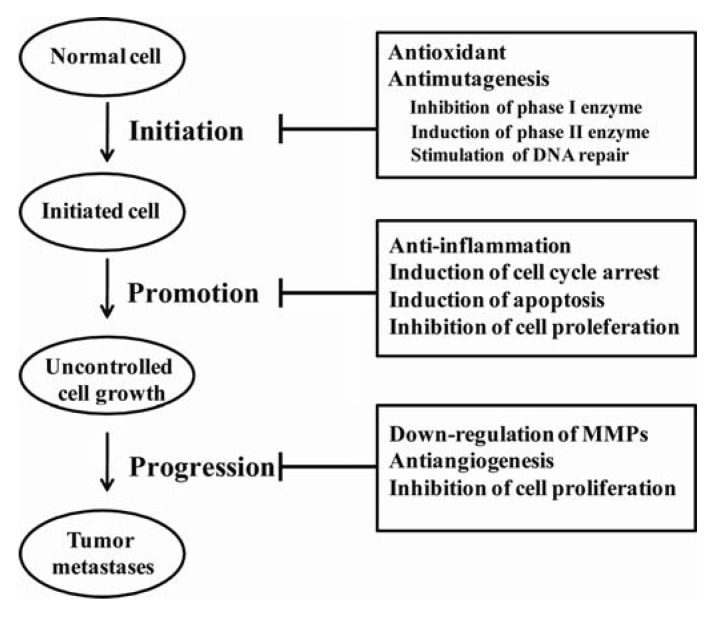
Potential anticancer mechanisms of plant phenolics during cancer development.

If the DNA damage escapes repair, it can lead to genetic mutation. The resulting somatic mutation in a damaged cell can be reproduced during mitosis, which given rise to a clone of mutated cells. Tumor promotion is a selective clonal expansion of the initiated cells to form an actively proliferating multi-cellular premalignant tumor cell population. It is an interruptible or reversible and long term process. During progression, premalignant cells developed into tumors through a process of clonal expansion. In the late stages of cancer development, invasion and metastasis happens, where tumor cells detach from the primary tumor mass, migrate through surrounding tissues toward blood vessels or lymphatic vessels, and create a second lesion. Metastasis is the major cause of cancer mortality. It is widely accepted that human cancer development does not occur through these discrete phases in a predictable manner, rather it is best characterized as an accumulation of alteration in cancer regulating genes [207], such as oncogenes, tumor suppressor genes, resulting in altered cellular processes, namely, decreased apoptosis, increased proliferation, and cell maturation and differentiation. The inhibitory effect of natural phenolics in carcinogenesis and tumor growth may be through two main mechanisms: 1) modifying the redox status and, 2) interfering with basic cellular functions (cell cycle, apoptosis, inflammation, angiogenesis, invasion and metastasis) [208].

#### 4.4.1. Antioxidant and prooxidant effect of phenolics on cellular redox status

ROS/RNS are constantly produced during normal cellular metabolism or by other exogenous means including the metabolism of environmental toxins or carcinogens, by ionizing radiation and by phagocytic cells involved in the inflammatory response. When the cellular concentration of oxidant species is increased to an extent that overcome the endogenous antioxidant defense system, oxidative stress occurs, leading to lipid, protein, and DNA damage. In addition, ROS, particularly H_2_O_2_, are potent regulators of cell replication and play an important role in signal transduction [209]. Hence, oxidative damage is considered a main factor contributing to carcinogenesis and evolution of cancer. Due to their ability to scavenge and reduce the production of free radicals and act as transition metal chelators, natural phenolic compounds can exert a major chemopreventive activity [208]. Indeed, it has been shown that natural polyphenols can inhibit carcinogen/toxin-induced cellular oxidative damage. For example, in nicotine-treated rat peripheral blood lymphocytes, ellagic acid effectively restored the antioxidant status and reduced DNA damage as well as lipid peroxidation [210]. A phenolic apple juice extract as well as its reconstituted polyphenol mixture (rutin, phloridzin, chlorogenic acid, caffeic acid and epicatechin) were shown to effectively reduce menadione-induced oxidative DNA damage and increasing of cellular ROS level [211]. Tea polyphenols [212] and other extensively studied polyphenols such as resveratrol [163,213], quercetin [214,215,216] were also showed to exert protective effects against cellular oxidative damage in different human cell lines.

UV radiation-induced ROS and oxidative stress is capable of oxidizing lipids, proteins, or DNA, leading to the formation of oxidized products such as lipid hydroperoxides, protein carbonyls, or 8-OHdG, which have been implicated in the onset of skin diseases including skin cancers [217,218,219]. Phenolic extracts, such as pomegranate-derived extracts [220], tea [221] and wine [222] extracts have been shown to reduce the oxidative damage of UV light in skin. Purified phenolic compounds such as anthocyanins [223], proanthocyanidin [224] and EGCG [225] were found to inhibit the UV-radiation-induced oxidative stress and cell damage in human keratinocytes.

On the other hand, *in vitro* studies also suggested that polyphenols may exert their inhibitory effects by acting as prooxidants on cancer cells. It has been reported that many polyphenols including flavonoids such as quercetin, rutin, apigenin, phenolics acids such as gallic acid, tannic acid, caffeic acid, as well as delphinidin, resveratrol, curcumin, gallocatechin and EGCG can cause oxidative strand breakage in DNA *in vitro* [226,227]. Furthermore, the cytotoxicity of quercetin and gallic acid on CaCo-2 cells and normal rat liver epithelial cells was partially reduced by antioxidant such as catalase [228]. Similar results have also been reported in oral carcinoma cell lines with EGCG [229]. These studies suggested that the antiproliferative effects of some polyphenol antioxidants on cancer cells are partially due to their prooxidant actions. However, it has been proposed that this oxidative property depends on the amount of dissolved oxygen in the test medium [230]. The oxygen partial pressure in a cell culture system (160 mmHg) is much higher than that in the blood or tissues (< 40 mmHg). It is not clear whether a similar mechanism could also occur *in vivo*.

#### 4.4.2. Interference of basic cellular functions by phenolics

Natural phenolics can affect basic cell functions that related cancer development by many different mechanisms. Firstly, in the initiation stage, phenolics may inhibit activation of procarcinogens by inhibiting phase I metabolizing enzymes, such as cytochrome P450 [231] and also facilitate detoxifying and elimination of the carcinogens by induction of phase II metabolizing enzymes such as glutathione S-transferase (GST), NAD(P)H quinine oxidoreductase (NQO), and UDP-glucuronyl-transferase (UGT) [232]. They may also limit the formation of the initiated cells by stimulating DNA repair [233,234].

Secondly, phenolics may inhibit the formation and growth of tumors by induction of cell cycle arrest and apoptosis. Malignant cells are characterized by excessive proliferation, inability to terminally differentiate or perform apoptosis under normal conditions, and an extended or immortalized life span. The regulation of cell cycle is altered in these cells. Thus, any perturbation of cell cycle specific proteins by phenolics can potentially affect and/or block the continuous proliferation of these tumorigenic cells. Natural phenolics have been reported induce cell cycle arrest at different cell phases: G1, S, S-G2, and G2 by directly down-regulating cyclins and cyclins-dependent kinases (CDKs) or indirectly inducing the expression of p21, p27 and p53 genes [158,235]. Moreover, some studies have shown that natural phenolics exhibit differential effect in cancer *versus* normal cells. For example, anthocyanin-rich extract from chokeberry was found to induce cell cycle block at G1/G0 and G2/M phases in colon cancer HT-29 cells but not in NCW460 normal colonic cells [236]. 

Apoptosis has been reported to play an important role in elimination of seriously damaged cells or tumor cells by chemopreventive or chemotherapeutic agents [232,237]. The cells that have undergone apoptosis have typically shown chromatin condensation and DNA fragmentation. They are rapidly recognized by macrophages before cell lysis, and then can be removed without inducing inflammation. Therefore, apoptosis-inducing agents are expected to be ideal anticancer drugs. Polyphenols have been found to affect cancer cell growth by inducing apoptosis in many cell lines such as the hepatoma (HepG2), the colon (SW620, HT-29, CaCo-2, and HCT-116), the prostate (DU-145 and LNCaP), the lung (A549), the breast (MCF-7), the melanoma (SK-MEL-28 and SK-MEL-1), the neuroblastoma (SH-SY5Y) and the HL-60 leukemia cells [238,239]. In many cases, apoptosis induced by polyphenols was caspase-3-dependent. The induction of apoptosis and/or inhibition of proliferation/survival by polyphenols has been reported to result from a number of mechanisms including inducing cell cycle arrest; blocking the extracellular regulated kinase (ERK), c-Jun *N*-terminal kinase (JNK), and P38 mitogen-activated protein kinase (MAPK) pathway; inhibition of the activation of transcription factors, NF-κB and activator protein-1 (AP1); suppression of protein kinase C (PKC); suppression of growth factor-mediated pathways [158,235]. For example, Afaq *et al*. showed that pomegranate fruit extract, rich in anthocyanins and hydrolysable tannins, protected against the adverse effect of both UVB-radiation in normal human epidermal keratinocytes *in vitro* [240] and 12-*O*-tetradecanoylphorbol-13-acetate (TPA) in CD-1 mouse skin *in vivo* [241], by inhibiting the activation of NF-κB and MAPK pathway. In addition, green tea polyphenols was found to protect against pentachlorophenol (PCP)-induced mouse hepatocarcinogenesis via its ability to prevent down-regulation of gap junctional intercellular communication (GJIC) which is strongly related to cell proliferation and differentiation [242]. Pure phenolic compound such as quercetin [243], resveratrol [244] were also found to block tumor promoter such as TPA-induced inhibition of GJIC.

One important aspect of carcinogenesis is recognized to be the involvement of inflammation. For instance, prostaglandins are mediators of inflammation and chronic inflammation predisposes to carcinogenesis. The over-expression of inducible cyclooxygenases (COX-2), the enzyme which catalyzes a critical step in the conversion of arachidonic acid to prostaglandins and is induced by pro-inflammatory stimuli, including mitogens, cytokines and bacterial lipopolysaccharide (LPS), is believed to be associated with colon, lung, breast and prostate carcinogenesis. Natural phenolics have been reported to inhibit transcription factors closely linked to inflammation (e.g., NF-κB) [245,246], pro-inflammatory cytokines release [245,247] and enzymes such as COX-2 [248,249], lipoxygenases (LOX) [250], inducible nitric oxide synthase (iNOS) [251] that mediate inflammatory processes, both *in vitro* and *in vivo* [252]. In many cases, polyphenols exhibit anti-inflammatory properties through blocking MAPK-mediated pathway. Furthermore, a few structure-activity studies have been conducted. For example, Hou *et al*. examined the inhibitory effects of five kinds of green tea proanthocyanidins on cyclooxygenase-2 (COX-2) expression and PGE-2 release in LPS-activated murine macrophage RAW-264 cells [248]. It was revealed that the galloyl moiety of proanthocyanidins appeared important to their inhibitory actions. Another study by Herath *et al.* suggested that the double bond between carbon 2 and 3 and the ketone group at position 4 of flavonoids are necessary for potent inhibitory effects on LPS-induced tumor necrosis factor-alpha (TNF-α) production in mouse macrophages (J774.1) [253]. 

Finally, natural phenolics such as green tea polyphenols (EGCG, GCG), grape seeds proanthocyanidins, hydrolysable tannins, genistein, curcumin, resveratrol, and anthocyanins, were found to suppress malignant cell migration, invasion and metastasis *in vitro* and *in vivo* [254,255,256,257,258,259]. The inhibition effect has been shown to be related to their ability to down-regulate the matrix metalloproteases (MMPs), namely, MMP-2 and MMP-9, as well as urokinase-plasminogen activator (uPA) and uPA receptor (uPAR) expression. In addition, phenolic compounds possess antiangiogenesis effects [260], which is an important aspect in the inhibition of tumor growth, invasion and metastasis. It has been reported that phenolic compounds such as ellagic acids, EGCG, genistein and anthocyanin-rich berry extracts inhibit tumor angiogenesis through down-regulation of vascular endothelial growth factor (VEGF), VEGF receptor-2 (VEGFR-2), platelet-derived growth factor (PDGF), PDGF receptor (PDGFR), hypoxia-inducible factor 1α (HIF-1α) and MMPs, as well as inhibition of phosphorylation of EGFR, VEGFR and PDGFR [235]. 

## 5. Conclusions

In summary, natural phenolics have been found to intervene at all stages of cancer development. In addition to their antioxidant action, the inhibition of cancer development by phenolic compounds relies on a number of basic cellular mechanisms, involving a spectrum of cellular basic machinery. Moreover, the extensive studies of this class of compounds will provide clues about their possible pharmaceutical exploration in the field of oncology.
